# Virtual Bioequivalence Assessment and Dissolution Safe Space Exploration for Fixed-Dose Metformin–Glyburide Tablet Using Physiologically Based Biopharmaceutics Modeling

**DOI:** 10.3390/pharmaceutics17101352

**Published:** 2025-10-20

**Authors:** Chenshuang Zhao, Chaozhuang Shen, Yumeng Xiao, Ling Wang

**Affiliations:** Department of Clinical Pharmacy and Pharmacy Administration, West China School of Pharmacy, Sichuan University, Chengdu 610064, China; zhaocs@stu.scu.edu.cn (C.Z.); 2023324050039@stu.scu.edu.cn (C.S.); 2022324050036@stu.scu.edu.cn (Y.X.)

**Keywords:** fixed-dose combination (FDC), physiologically based biopharmaceutics modeling (PBBM), virtual bioequivalence (VBE), sensitivity analysis, safe space, metformin, glyburide, PK-Sim

## Abstract

**Background/Objectives**: Fixed-dose combinations (FDCs) hold significant clinical value for the management of hypertension, diabetes and other chronic diseases. However, since the complexity of formulations, generic compounds require both in vitro pharmaceutical equivalence and in vivo bioequivalence (BE) for each active pharmaceutical ingredient (API). Physiologically based biopharmaceutics modeling (PBBM) not only bridges in vitro drug properties to in vivo pharmacokinetics but effectively assesses the impact of formulations on systemic exposure. This study was aimed at developing a PBBM for metformin–glyburide FDC and investigating its clinically relevant quality specifications. **Methods**: PK-Sim^®^ software (Version 11.3) was used to establish a PBBM for a metformin–glyburide FDC. Sensitivity analysis identified critical parameters and guided design of virtual populations. Subsequently, virtual bioequivalence (VBE) was assessed between both reference and test formulations, and BE-ensuring dissolution space was explored by the change in dissolution characteristics. **Results**: The in vivo behavior of products was successfully captured by the developed model. Sensitivity analysis indicated that systemic exposure was primarily sensitive to gastrointestinal (GI) pH and transit times. VBE analysis confirmed BE between the reference and test formulations. The dissolution safe space for the FDC was defined as the concurrent achievement of ≥ 50% dissolution within 25 min for metformin and between 35 and 170 min for glyburide, which constituted equivalent specification. **Conclusions**: The PBBM developed in this study systematically evaluated the VBE of metformin–glyburide FDC, optimized the acceptance criteria for traditional in vitro dissolution testing, and thereby explored its clinically relevant quality specification.

## 1. Introduction

Fixed-dose combination (FDC) preparations typically contain no less than two active pharmaceutical ingredients (APIs) within a single formulation [1]. These formulations offer several advantages, including improved patient compliance, reduced healthcare costs, simplified clinical decision-making, and a diminished risk of adverse drug reactions. These benefits are especially valuable in conditions like diabetes, which often require long-term combination therapy, making FDCs an attractive strategic option for patients facing polypharmacy. Over the past two decades, the approval of FDCs has steadily increased, accounting for a median of 7–10 new approvals annually, or approximately 9% of new drug applications [2]. The therapeutic scope of FDCs has also diversified considerably. They are abundantly available for comorbid conditions such as hypertension and diabetes, pain and muscle relaxation, and various infectious diseases. Furthermore, FDCs within the same pharmacological category are widely used to treat single ailments, including tuberculosis, Parkinson’s disease, and urinary tract infections, underscoring their broad global application and acceptance [3].

Diabetes represents a major global chronic disease burden. According to the International Diabetes Federation (IDF), approximately 589 million adults aged 20–79 were living with diabetes worldwide in 2024 [4]. International treatment guidelines consistently recommend metformin as the first-line pharmacological therapy. For a significant proportion of patients who fail to achieve adequate glycemic control with metformin monotherapy, combination therapy with agents possessing complementary mechanisms of action is recommended [5,6]. This clinical need makes FDC strategies particularly valuable for the long-term, often lifelong, management of diabetes, as they simplify regimens and potentially improve adherence. Consequently, numerous antihyperglycemic FDCs have been developed. Among these, the combination of metformin and glyburide is one of the earliest and most widely used FDCs. 

As a classic FDC hypoglycemic agent, metformin–glyburide tablets exhibit superior efficacy in glycemic control. The metformin–glyburide FDC tablet is indicated for patients with T2DM who have not achieved adequate glycemic control through diet and/or exercise alone, or with monotherapy using either sulfonylureas or metformin. Glyburide, a representative second-generation sulfonylurea, lowers blood glucose primarily by stimulating insulin secretion from pancreatic β-cells. Metformin, an insulin sensitizer, reduces glucose levels through multiple mechanisms, including inhibition of hepatic gluconeogenesis, decreased intestinal glucose absorption, and enhanced peripheral glucose uptake and utilization. This combined therapy not only effectively regulates blood glucose levels but also improves overall glucose metabolism in patients. A meta-analysis has reported that metformin is associated with faster glycemic control and reduced postprandial glucose concentrations, whereas glyburide is linked to reductions in fasting plasma glucose and HbA1c. Moreover, a bioequivalence study was conducted comparing two formulations of metformin hydrochloride/glyburide tablets (500 mg/5 mg) under both fasting and fed conditions, which confirmed the bioequivalence and safety of the two formulations [7,8]. However, the marked differences in the physicochemical properties of metformin and glyburide present considerable challenges to formulations. Metformin, classified as a Biopharmaceutics Classification System (BCS) Class III drug, possesses high solubility but low permeability, while glyburide as a BCS Class II drug, demonstrates low solubility and high permeability. The differences in chemical and physical properties between the two active pharmaceutical ingredients necessitate additional considerations during the development of the FDC product. 

Despite appearing as combinations of more than two single-ingredient drugs, the development of FDC products presents inherent complexities, which may result in APIs failing to reach the target site at the desired concentration predictably and reproducibly. An example is that the dissociation of one active ingredient may affect the stability of others or even result in the generation of new degradation products, particularly for APIs unstable in gastrointestinal environments or requiring specialized formulation processes. Consequently, national and regional regulatory guidelines for the in vivo bioequivalence (BE) assessment of FDCs are considerably more stringent than those for the in vivo BE assessment of single-entity formulations, and require demonstrating BE for all the medication components in a FDC product. The entire combination fails when one component fails to demonstrate BE [9,10,11].

Advances in drug modeling technology have contributed to the growing prevalence of model-informed drug development (MIDD). These approaches offer several benefits, including a reduced need for subject exposure, lower development costs, and accelerated market entry for novel therapeutics. Notably, the application of physiologically based biopharmaceutics modeling (PBBM) has gained significant traction in the development of generic drug in recent years. As an advanced extension of the physiologically based pharmacokinetic (PBPK) model, PBBM is specifically tailored for biopharmaceuticals. The PBPK model incorporates physiological and drug parameters and predicts the in vivo exposure levels of the drug [12,13,14,15,16], while PBBM focuses on how in vivo processes are influenced by formulation properties [17,18,19]. PBBM integrates the characteristics of various formulations with human physiological properties, especially the gastrointestinal state, and encompasses all the quality attributes of APIs and drugs affecting in vivo performance. In particular, PBBM is useful in developing prescriptions, assessing biopharmaceutical risks, comparing formulation dissolution profiles, evaluating food effects and facilitating bio-waivers during the process of generic drug development [19,20].

PBBM enables the integration of formulation characteristics with the in vivo processes of metformin–glyburide FDC. The aim of this study was to develop a robust model and further investigate: (1) key sensitivity factors influencing drug absorption and systemic disposition; (2) virtual bioequivalence in populations under varying gastrointestinal physiological conditions; (3) the establishment of a safe dissolution space ensuring bioequivalence.

## 2. Materials and Methods

### 2.1. Chemicals and Reagents

The metformin–glyburide tablets investigated were reference (Glucovance^®^, Merck Sante S.A.S, Lyon, France) and test tables. The specification used was 500 mg/2.5 mg IR tablets containing 500 mg metformin hydrochloride and 2.5 mg glyburide.

### 2.2. Software

PK-Sim^®^ and Mobi^®^ (Version 11.3, www.open-systems-pharmacology.org) were utilized to develop models. R (Version 4.4.1, The R Project for Statistical Computing, www.R-project.org) was adopted to compare and analyze BE between test and reference products.

### 2.3. In Vitro Dissolution Tests

The tests were carefully designed based on the physicochemical properties of the drugs and in accordance with relevant guidelines.

The paddle method was employed to determine the dissolution profiles of the tablets in 1000 mL of three different dissolution media: hydrochloric acid solution (pH 1.2, 0.1 mol/L), acetate buffer (pH 4.5, 0.2 mol/L, buffer capacity: 0.1 mol/L⋅pH^−1^), and phosphate buffer (pH 6.8, 0.2 mol/L, buffer capacity: 0.1 mol/L⋅pH^−1^) at 37 °C and 50 rpm. For glyburide, which has poor solubility, the sink condition was achieved by adding the surfactant sodium lauryl sulfate (SDS) to the medium. Samples were withdrawn at 5, 10, 15, 30, 45, and 60 min for metformin and collected at 5, 10, 15, 30, 45, 60, 90, 120, 180, and 240 min for glyburide. All the samples were filtered through a 0.45 μm cellulose membrane, and the concentration of metformin and glyburide were detected by UV and HPLC-UV, respectively. The detailed methodological considerations and dissolution profiles with f2 factor are summarized in Supplementary Material Section S1. 

### 2.4. In Vivo Pharmacokinetic Studies

**Figure 1:**
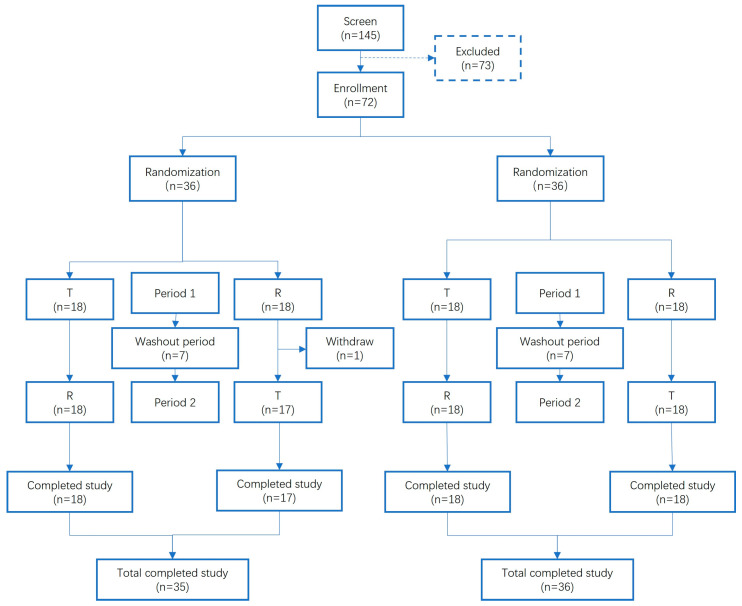
Study design and subject flow. The diagram illustrates the participant flow for the entire bioequivalence study. The study included both fasted and fed treatment arms in a crossover design. For the purpose of validating the model developed in this work, only the pharmacokinetic data from the fasted treatment arm were used.

The data on plasma concentrations were obtained from an open-label, two-part and two period crossover BE study performed on 72 healthy Chinese participants (Figure 1). Enrolled participants were randomly assigned to two groups using a computer-generated randomization schedule. Blood samples were collected at 22 time points: pre-dose (0 h) and 0.25, 0.5, 1, 1.5, 2, 2.5, 3, 3.5, 4, 4.5, 5, 6, 6.5, 7, 7.5, 8, 10, 12, 24, 36 and 48 hours post-dose. Drug concentrations in plasma were quantified using validated HPLC-MS/MS methods. The data were used for the model development and validation. Although the clinical study included both fasted and fed conditions, only the data from the fasted treatment arm were used for model validation.

The study was conducted in accordance with the Declaration of Helsinki, and approved by the Institutional Ethics Committee of the First Affiliated Hospital of Shantou University Medical College before implementation (protocol code A-2023-016-CS and protocol date 7 October 2023). All participants signed written informed consent before inclusion in the experimental group. 

### 2.5. In Silico Model Development

PK-Sim^®^ was applied to develop metformin and glyburide PBPK models, respectively. The PBPK models incorporated PK-Sim^®^ integrated population database and employing a bottom-up approach to systematically combine compound-specific physicochemical properties with human physiological system parameters [21]. 

#### 2.5.1. Physiology and Pharmacokinetic Parameters

The Asian population (Tanaka, 1996) embedded in the software was selected as the demographic configuration for model parameterization, which was consistent with Supplementary Table S1. In the base PBPK framework, system-specific physiological parameters including gastrointestinal characteristics were retained at default values. 

The physicochemical parameters of metformin and glyburide were acquired from the literature and databases via extensive efforts to collect data. The parameters of these two drugs associated with the process of absorption, distribution, metabolism and excretion in the human body were obtained from three sources, literature data, model assumption and model optimization, as shown in Table 1. 

#### 2.5.2. Model Development

The pharmacokinetic profiles of metformin and glyburide were characterized in a thorough way to construct PBPK base models for each drug. The physicochemical parameters collected in Section 2.5.1 were first input into the respective building blocks.

Metformin is a typical BCS Class III drug, characterized by high solubility but low permeability. It dissolves rapidly and is primarily absorbed in the small intestine upon administration. In the small intestine, metformin is transported through the plasma membrane monoamine transporter (PMAT) located at the apical membrane of the intestinal mucosal epithelium [24,26]. For the purpose of accurately representing this critical transport, the distribution of PMAT incorporated into the model was quantified using RT-PCR data, and the specific transport kinetics were described by the Hill’s equation. The predicted PMAT Vmax was optimized to a final value of 53.36 1/min. Metformin demonstrates negligible plasma protein binding and its in vivo elimination does not undergo hepatic metabolism or biliary excretion. Instead, it is mainly excreted unchanged in urine, with renal tubular secretion being the predominant route [27]. To describe this elimination process, the renal tubular secretion panel of PK-Sim^®^ was utilized, and the process was defined with the Michaelis-Menten equation. The optimized TS_max_spec_ for this process was 68.42 μmol/L/min.

Unlike metformin, glyburide is classified as a BCS Class II drug and typified by low solubility and high permeability. Therefore, its in vivo absorption is limited by the rate and extent of dissolution. The solubility of glyburide is pH-dependent—extremely low in acidic environments like gastric fluid, where glyburide primarily exists as undissociated molecules, and slightly increased in neutral to weakly alkaline environments like in the small intestine. The reference pH for solubility was optimized in the PBPK model in order to reflect this behavior. Glyburide is 99.9% bound to protein in plasma with > 98% accounted for by binding to serum albumin [28]. Glyburide is primarily eliminated in vivo via hepatic biotransformation, with the subsequent biliary and urinary excretion of its metabolites. Given the negligible hypoglycemic activity of glyburide metabolites, the model used in this study focused exclusively on the parent compound. Total hepatic plasma clearance in PK-Sim^®^ was used for describing the elimination process, and the final fitted specific clearance value was determined to be 4.14 L/mL. 

For the purpose of reflecting the differences across formulations, the model was developed by incorporating a formulation-specific release process. The dissolution behavior of the formulation was empirically depicted using the Weibull function, which is widely employed for modeling drug release kinetics. The Weibull equation is given as follows:

(1) $$F(t)=1-exp(\frac{-{\left(t-T_{lag}\right)}^{b}}{a})$$

where *F(t)* represents the fraction of drug released at time *t*; *a* stands for the scale parameter that defines time after the start of dissolution when 50% of the administered dose is dissolved; *T_lag_* denotes the location parameter and represents lag time before the onset of drug release; and *b* indicates the shape parameter, which determines the curvature of the release profile. The application of the Weibull function requires defining these parameters in PK-Sim^®^ in advance. The calculation and verification of these parameters from in vitro experiments was performed with ddsolver [29], and the calculations results were input into the building block. 

After the establishment of structural models for the two drug components, the parameter identification function in PK-Sim^®^ was employed. The Monte Carlo algorithm was employed to optimize models.

#### 2.5.3. Model Validation

A virtual population corresponding to 100 cases was constructed for each simulation in accordance with to the demographic information (Supplemental Table S1) of human BE study subjects.

The model was validated in a multidimensional manner. Firstly, the reported mean blood concentration profiles were compared with the predicted population PK profiles for evaluating the similarity of model fit. Secondly, the relevant pharmacokinetic parameters representing systemic exposure levels, i.e., C_max_ and AUC, were systematically evaluated for Fold Error (FE) and Prediction Error (PE) (Equations (2) and (3). FE ≤ 2 and PE ≤ 20% indicated the good predictive ability of the model.

(2) $$PE=\frac{|\mathrm{predicted}-\mathrm{observed}|}{\mathrm{observed}}$$

(3) $$FE=\frac{predicted}{observed}$$

### 2.6. Virtual Bioequivalence Study Design

#### 2.6.1. Assessment of Parameter Sensitivity

Sensitivity analyses were performed separately on the models developed in Section 2.5 to assess whether pharmacokinetic parameters (C_max_, T_max_ and AUC) were sensitive to each parameter in the model. The purpose of this part of work was to screen out the parameters with the most impact on the model. In this way, appropriate variants can be added to the virtual population. The sensitivity of the PK parameter to the input parameter was calculated as Equation (4):

(4) $$S_{ij}=\frac{∆PK_{j}}{∆P_{i}}\cdot \frac{P_{i}}{PK_{j}}$$

where S_ij_ represents sensitivity, ΔPK_j_ stands for the change in PK_j_ parameter; P_i_ denotes original model parameter values. The purpose of the equation is to quantify how a small change in an input parameter affects the output result, such as pharmacokinetic parameters like AUC or Cmax. The sign of the calculated sensitivity coefficient indicates the direction of the effect, while the magnitude reflects the strength of the impact. For example, a coefficient of −1.0 indicates that a 10% increase in the input parameter would lead to a proportional 10% decrease in the pharmacokinetic parameter. Conversely, a coefficient of +0.5 implies that a 10% increase in the input parameter would result in only a 5% increase in the pharmacokinetic parameter. 

Parameters with sensitivity scores < 0.05 were excluded from this study. GI tract-related parameters affecting absorption were selected as main variants in the population. The encompassed pH at various sites in the GI tract (including stomach, duodenum, jejunum, ileum, colon and rectum), gastric emptying time, pH in the fasted state, undisintegrated portion of the GET, and large and small intestinal transit time. By setting the variation in gastrointestinal parameters in the population, the formulation would be absorbed in different states. Thus, the demonstration of the differences between formulations.

#### 2.6.2. Virtual Population Construction and BE Analysis

Virtual populations were constructed for virtual BE studies in PK-Sim^®^. Variants were set for the virtual population on the basis of the sensitivity analysis results. The variation ranges for delineation parameters were obtained from Mobi^®^ and the literature (Table S2). The population with the set range of variants was subjected to the random generation of GI parameters by R. Other settings were aligned with those portrayed in Section 2.4. Virtual bioequivalence (VBE) assessment was also conducted using a 2 × 2 crossover study design. Based on the established workflow, the pre-designed virtual population was randomly assigned to two sequences (TR and RT). Each sequence was further stratified into two subgroups representing different gastrointestinal (GI) conditions (GI1 and GI2). For example, the TR sequence was divided into P1 (GI1) and P2 (GI2), which were administered the test and reference formulations, respectively. In the first sequence, one group received the reference product followed by the test product, whereas in the second sequence, the order was reversed, with the test product administered first, followed by the reference product (Table S3). The BE of the final simulation was compared between the reference and the test preparation, and the analysis of bioequivalence was also achieved by R.

### 2.7. Development of Safe Space

The safe dissolution space is a concept delineating the in vivo BE range through in vitro dissolution profiling [17]. In this study, the dissolution space was generated by the modulation parameters in the Weibull model (Equation (1)), which characterizes drug dissolution through three parameters: dissolution time (*a*), shape factor (*b*), and lag time (*T_lag_*). In light of the optimized dissolution parameters of the test formulation (Table 1), time to 50% dissolution was systematically varied to alter dissolution profiles, which thereby simulated distinct formulation variants. Through the systematic change in dissolution parameters in the model and the subsequent incorporation of the modified parameters, new population PK profiles were generated, and virtual population BE analysis was conducted using R 4.3.1. This iterative process continued to achieve 90% CI of C_max_ and AUC ratios ranging between 80.00% to 125.00%.

## 3. Results

### 3.1. PBPK Models and Validation

**Figure 2:**
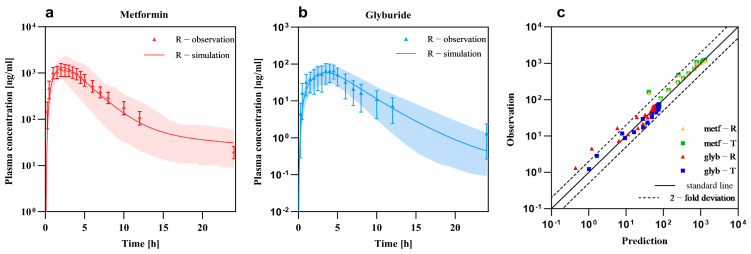
Model fitting results and GOF plots: (**a**,**b**) represent the model fitting outcomes for the reference formulations of metformin (**a**) and glyburide (**b**); (**c**) depicts the goodness-of-fit (GOF) plot after simulation, where the solid line represents the standard line and the dashed line indicates the two-fold error line.

The PBPK models of metformin and glyburide were developed and validated as described in Section 2.5. A virtual Asian population that comprised 100 subjects aged 18-60 with BMI ranging from 19 to 27 was later constructed for model validation. PK-Sim^®^ was utilized to develop the PBPK model. The predicted pharmacokinetic parameters significantly aligned with the observed ones, and the generated simulated profiles matched the observed data closely (Figure 2). In all cases, the predicted error did not exceed 0.2, which was indicative of excellent model performance (Table 2). 

### 3.2. Virtual Bioequivalence Study

#### 3.2.1. Sensitivity Analysis

**Figure 3:**
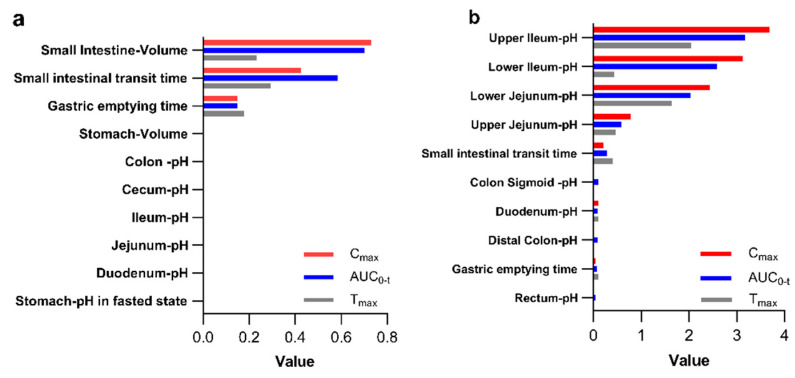
Global sensitivity analysis for key pharmacokinetic parameters of metformin–glyburide tablets: (**a**) displays the results for metformin component and (**b**) for glyburide component.

Sensitivity analysis is a critical component of PBPK modeling [30], which was conducted in both metformin and glyburide models. For metformin, key human factors affecting C_max_, AUC and T_max_ (sensitivity ≥ 5%) were small bowel transit time, gastric emptying time and large bowel transit time in descending order. For glyburide, GI pH exhibited greater influence, and jejunal/ileal pH, small bowel transit time, duodenal pH and gastric emptying time showed descending sensitivity. Notably, colonic pH impacted AUC_0-t_ for glyburide but demonstrated zero sensitivity for C_max_. Comprehensive sensitivity results are presented in Figure 3.

#### 3.2.2. Virtual Population

A virtual population was established based on the sensitivity analysis results, and a virtual BE study was performed in this population. The inter-individual variability (IIV) or between-subject variability (BSV) between individuals of a population was stored in population database of PK-Sim^®^. Based on the sensitivity analysis results in Section 3.2.1, a representative virtual population was constructed to simulate within-subject variability (WSV) within the real population. WSV parameters ranged according to the variability specified in Table S2 and were further defined for two distinct GI states. Afterwards, a 2 × 2 crossover study group was established for systematically conducting a VBE study (Table S3).

#### 3.2.3. Virtual Bioequivalence Analysis

**Figure 4:**
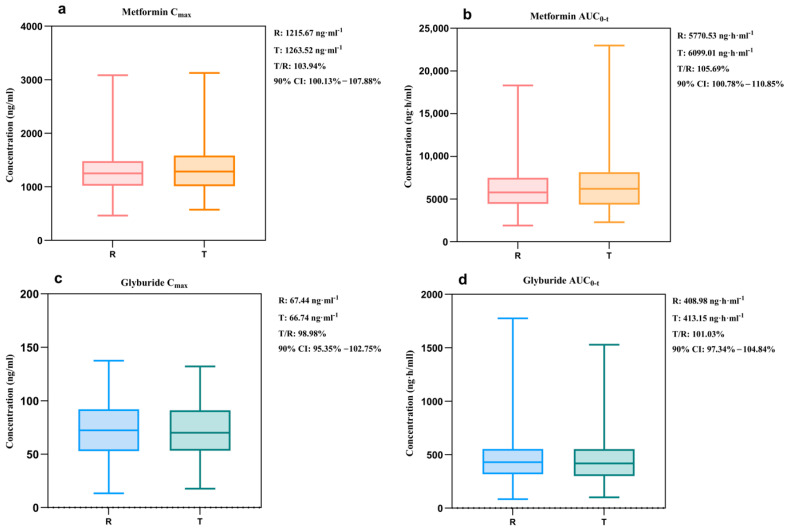
Pharmacokinetic parameter plots of the virtual population. (**a**,**b**) C_max_, AUC_0-t_ of metformin with different sequences, respectively. (**c**,**d**) C_max_, AUC_0-t_ of glyburide with different sequences, respectively.

The relevant pharmacokinetic parameters of the VBE model were analyzed, and the results are displayed in Figure 4. The results showed that the predicted C_max_ for the test and reference formulations of metformin were 1263.52 ng/mL and 1215.67 ng/mL, respectively, and AUC_0-t_ was 6099.01 ng/h/mL and 5770.53 ng/h/mL. The test and reference formulations of glyburide predicted C_max_ of 66.74 ng/mL and 67.44 ng/mL with AUC_0-t_ of 413.15 ng/h/mL and 408.98 ng/h/mL, respectively. The 90% confidence intervals fell within 80–125% of each other within the limits. The 90% CI for geometric mean ratios all fell within the 80–125% acceptance range, for metformin C_max_ 90%CI was 100.13–107.88% and AUC was 100.78–110.85%, while for glyburide C_max_ 90%CI was 95.35–102.75% and AUC was 97.34–104.84%. Hence, both the drug components of the combination were bioequivalent in the constructed virtual population.

### 3.3. Dissolution Safe Spaces

**Figure 5:**
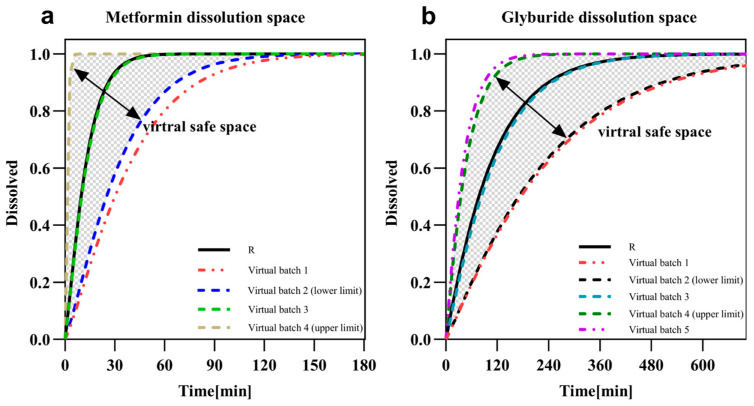
Expected bioequivalent dissolution safe space for metformin (**a**) and glyburide (**b**). Gray shaded areas delineate the dissolution curves of metformin and glyburide where bioequivalence can be achieved under the fastest and slowest conditions. Virtual batches represented by dashdot lines failed to achieve bioequivalence with the reference batch, including the red dashed lines in Figure a (Virtual Batch 1) and the red and purple dashed line in Figure b (Virtual Batch 1 and 5).

The dissolution space and associated failure boundaries for the compound were defined by adjusting dissolution time in Weibull function. The subsequent analysis of safe dissolution spaces demonstrated that bioequivalence could be achieved when 50% dissolution occurred within 25 min for compound metformin and within 35 to 170 min for compound glyburide (Figure 5, Table 3 and Table 4). 

## 4. Discussion

In the present study, a PBPK model was developed for metformin–glyburide tablets, and formulation dissolution kinetics were simulated using the Weibull function. Sensitivity analysis was followed by the establishment of a virtual population. Then, the virtual bioequivalence between the test and reference formulations was compared. Finally, a safe dissolution space was constructed to predict the in vivo performance of different formulations and their variations. 

PBPK modeling typically requires numerous input parameters, but the impact on outputs is often unclear. Sensitivity analysis quantifies the influence of individual parameter variations on model results [30]. In the current study, sensitivity analysis for each API in the combination product revealed that metformin absorption was predominantly sensitive to gastrointestinal transit time. However, glyburide absorption was affected by both gastrointestinal pH and transit time. These findings align with the known properties of the drugs. The high solubility and low permeability of metformin are intrinsically determined by its molecular structure. The drug contains a strongly polar guanidine group, exhibits an extremely low log P value (−1.43) [22], and behaves as a strong base (pKa ≈ 11.5) [23]. Consequently, metformin exists predominantly in a hydrophilic, cationic form throughout the physiological pH range of the gastrointestinal (GI) tract, resulting in its characteristic high solubility and low permeability. Following oral administration, the tablet disintegrates in the gastric fluid within minutes due to the action of disintegrants and GI motility, breaking down into fine particles or powder. This process significantly increases the surface area of the drug. Owing to its high hydrophilicity, metformin rapidly dissolves in the aqueous GI environment, forming a drug solution. However, its poor solubility in lipid environments hinders passive diffusion across the intestinal epithelium. Absorption primarily relies on specific membrane transporters, whose limited number, function, variable distribution, and significant inter-individual variability collectively contribute to the low intestinal permeability of metformin. Dissolution study results (Supplementary Material Section S1) confirm that both the reference and test formulations of the compound tablet release over 85% of the drug within 30 min, meeting the criteria for rapid release. Furthermore, the solubility of metformin in pH 6.8 medium can reach 350.9 g/L [23]. Therefore, dissolution is not the rate-limiting step for the absorption of metformin in vivo; the API dissolves rapidly and completely in the GI fluid. However, due to its slow intestinal permeability, dissolved drug accumulates in the GI tract. For this reason, transit time becomes the critical determinant for absorption. A longer residence time in the intestine allows for more complete absorption. For glyburide, the low solubility and high permeability are similarly dictated by its molecular structure. Its high lipophilicity (log P 3.75) results in low equilibrium solubility in water. Molecules on the surface of drug particles require considerable time to enter the solution and form a saturated layer, making dissolution rate and solubility the primary rate-limiting factors for its absorption. Additionally, the sulfonylurea group in glyburide’s structure contains a nitrogen-bound hydrogen atom with notable acidity, rendering glyburide a weak acid (pKa ≈ 5.3) [25]. According to the pH-partition hypothesis, a weak acid exists in equilibrium between its molecular (unionized) and ionic forms in solution, with the ratio determined by the environmental pH and the drug’s pKa. In its unionized form (predominant at low pH), glyburide is highly lipophilic but exhibits very poor aqueous solubility. Conversely, the ionized form (predominant at higher pH) demonstrates significantly enhanced water solubility. Therefore, slight variations in GI pH can markedly alter the dissolution kinetics of glyburide, directly impacting its absorption rate and extent. This mechanism aligns perfectly with the sensitivity analysis results, which indicated that the pH in various segments of the small intestine and the transit time significantly influence the in vivo performance of glyburide. Based on the above analysis: (1) In the stomach (pH ~ 1.2, absence of bile salts, low volume), where the pH is far below glyburide’s pKa, the drug exists almost exclusively in its unionized form. Consequently, solubility is extremely low, and negligible dissolution or absorption occurs. (2) In the small intestine (pH ~ 6.0–6.8, high bile salt concentration, large volume), the elevated pH favors ionization, significantly increasing intrinsic solubility. Furthermore, the high concentration of bile salts forms micelles, providing potent micellar solubilization for glyburide. This makes the small intestine the primary and most efficient site for its absorption. (3) In the colon (pH ~ 6.5–7.5, near absence of bile salts, low fluid volume, high viscosity), while the pH conditions are favorable for ionization, the critical lack of bile salts eliminates the micellar solubilization effect. Combined with the extended time required to reach the colon and the unfavorable fluid dynamics, the colon is not a significant absorption site for glyburide. Two distinct PK-Sim^®^ population variant sequences were used to generate virtual population (Table S3). For population segmentation across different dosing cycles within each sequence, differentiated GI physiological parameters, primarily GI transit time and segment-specific pH, were incorporated based on key sensitivity analysis findings. Notably, sensitivity analysis identified the significant effects of elimination-phase parameters (renal tubular secretion Km and TSmax_spec for metformin, and hepatic clearance for glyburide), but these parameters were held constant across cycles within individual subjects. This design aligned with the study objective of specifically assessing differences between test and reference formulations. With regard to oral solid dosage forms, formulation differences primarily manifest during drug absorption. Variability in the absorption environment was simulated and amplified by varying GI parameters, which thereby maximized the detection of formulation differences under physiologically fluctuating conditions. In contrast, elimination-phase parameters characterized post-absorption systemic drug disposition after absorption. These are governed by the API’s chemical structure and subject physiology/pathology of the API, rather than formulation characteristics. Introducing variability here would add unnecessary noise and obscure the specific formulation differences under investigation. However, in designing the virtual population, we introduced interindividual variability to drug-sensitive parameters in order to maximize the differentiation between the test and reference formulations. However, the virtual subjects in this study were modeled as healthy individuals, whereas the FDC product under investigation is intended for use in patients with diabetes—particularly those with long-standing disease. Some physiological parameters in diabetic patients differ significantly from those of healthy individuals. Under such altered physiological conditions, whether the two formulations can still achieve bioequivalence is a more clinically relevant question. This will be a key focus of our future research. 

Model validation plays a critical role in the modeling process, as effective validation ensures the credibility and reliability of the model. In this study, external validation was primarily employed by comparing model-simulated results with independent clinical datasets. Goodness-of-fit and prediction error analyses were used to evaluate the consistency between simulated and observed data. In addition, model-predicted VBE outcomes were compared with key pharmacokinetic parameters of the drug itself, such as bioavailability, AUC and Cmax. These are commonly accepted approaches in PBPK model validation. However, these methods also have inherent limitations. First, as a bottom-up modeling approach, PBPK modeling involves numerous parameters, many of which are derived from in vitro experiments and may require optimization during model integration [21,31]. The validation of such parameters is challenging. One commonly adopted strategy is multi-dose validation using clinical datasets under different dosing conditions to constrain the model’s applicability domain. This approach has been explored in our earlier work, though it is not presented in the current manuscript. Second, regarding the validation of the dissolution space, we conducted dissolution safe space predictions only after verifying the robustness and predictability of the model in prior work. Regulatory agencies (e.g., FDA, EMA) often require applicants to submit clinical data from non-bioequivalent (non-BE) batches to verify the model’s predictive accuracy [20]. If the model is able to correctly distinguish non-BE batches, its predictive capability would be further substantiated.

FDCs necessitate more complicated study designs than single-agent formulations. Ideally, the dissolution profiles of each ingredient in an FDC should closely resemble those of the corresponding single-agent formulations. However, discrepancies are often observed in practice. It has been observed that for glyburide single-ingredient drug, the T_max_ typically occurs around 2 hours [32,33,34]; however, in combination formulations, the T_max_ is significantly delayed. The first reason is that physicochemical properties of metformin and glyburide are vastly different. Different ingredients may also interact and affect drug release. To overcome the issue of poor solubility, glyburide single-ingredient drugs are typically formulated using techniques such as micronization to enhance rapid disintegration and dissolution [35,36], thereby accelerating absorption and resulting in a relatively short T_max_. In contrast, although metformin is rapidly absorbed, its formulation often requires modulation of the release rate and site within the gastrointestinal tract in order to mitigate common gastrointestinal adverse effects (e.g., diarrhea, nausea) and to maintain stable plasma drug concentrations. The co-formulation of these two drugs, which differ markedly in their physicochemical and pharmacokinetic properties, poses a formulation challenge. If the combination tablet is designed for rapid disintegration to favor glyburide release, it may lead to immediate release of metformin, potentially triggering pronounced gastrointestinal reactions. Conversely, controlling the release of metformin to minimize side effects may inadvertently delay the release of glyburide. Given the critical importance of gastrointestinal tolerability for patient adherence, the combination formulation is designed to modulate the overall release kinetics, thereby reducing the release and dissolution rate of glyburide compared to its immediate-release monotherapy counterpart. Thus, CPPs and CMAs need to be carefully selected during the design phase to ensure the proper release and dissolution of different ingredients. The second reason is that the formulation contains 500 mg of metformin and only 2.5 mg of glyburide. The hundreds fold dose difference between these two APIs renders it extremely challenging to ensure that low dose glyburide is uniformly distributed in large dose metformin [37]. This is a core difficulty in developing compounded formulation. The inherent difficulty stems from the need to achieve a homogeneous distribution of a minute quantity of the API within a much larger mass of excipients during powder blending and subsequent processing steps. Any inadequacy in mixing or subsequent particle segregation could lead to substantial tablet-to-tablet content variability, critically compromising the safety and efficacy of the final product. To address this challenge, a systematic strategy integrating formulation optimization and process control was employed. From a formulation perspective, the approach included optimizing the composition ratio, carefully controlling the particle size distribution of both the API and excipients, and incorporating suitable binders and glidants [35,37,38]. These measures aimed to enhance blend homogeneity by improving powder flow properties and reducing disparities in particle characteristics between glyburide and the excipients. Regarding processing, critical parameters were rigorously controlled. This involved optimizing the blending equipment and parameters (such as blending time and rotational speed) and strictly controlling conditions during subsequent unit operations like drying and tablet compression (e.g., temperature, pressure, and speed) to prevent segregation and ensure consistent product quality.

Many factors increase the likelihood of or even the failure of dissolution profile disparities, between test and reference compounded formulations during in vitro testing. Notably, such in vitro failures may not preclude in vivo bioequivalence. To address this, a safe dissolution space was constructed for compounded preparations. Within this defined space, formulations with different dissolution behaviors are all bioequivalent to the reference preparation. However, only batches of compounded formulations satisfying the safety space for both APIs are considered equivalent. It should be emphasized that this space is based solely on the characteristics of the compounded formulation, not just a combination of two individual drugs. 

Current modeling approaches that bridge in vitro dissolution with in vivo performance encompass both non-mechanistic (e.g., Weibull) and mechanistic methods (e.g., Johnson, Wang–Flanagan, Z-factor model). Mechanistic models such as those developed by Johnson, Wang–Flanagan, and the Z-factor approach are often more mechanistically driven, aiming to describe specific physical processes such as diffusion and erosion. The absorption of BCS Class II drugs, like glyburide, involves complex and dynamic processes including dissolution, precipitation, nucleation, and re-dissolution, and may also involve interactions with luminal components such as bile acids. However, in complex systems such as the FDC formulation studied in this work, the dissolution process diverges significantly from that of single-API formulations. In such cases, not only must the aforementioned factors be considered, but the dissolution behavior of each API may also be altered due to formulation-specific interactions. Moreover, incorporating all these parameters into a mechanistic model presents two major challenges: (1) reliable and accurate data for these parameters are often unavailable, particularly for combination products; (2) validating such a highly complex model is itself a significant challenge. In addition, the selected dissolution model must be capable of simultaneously and appropriately describing the dissolution kinetics of both APIs in the FDC. Due to this complexity, the Weibull function was employed to empirically simulate the dissolution kinetics of glyburide. The Weibull model is an empirical approach whose main advantage lies in its flexibility. It does not assume a predefined dissolution mechanism, but instead uses a shape parameter (β) to flexibly describe various dissolution profiles, including exponential, sigmoidal, and parabolic curves. This allows for a superior fit to experimental data. Furthermore, the Weibull model is readily integrated within the PK-Sim^®^ platform. Our primary objective was to establish a mathematical model that can accurately capture the entire dissolution process to provide a reliable input for subsequent PBPK simulations. In this context, the Weibull model has been widely demonstrated to be highly effective. 

Formulation variants spanning diverse release kinetics were generated by modulating model dissolution parameters. Subsequently, VBE studies were conducted against the reference formulation, and the dissolution safe space was determined. This helps spot potential quality issues, as dissolution behavior outside this space may indicate quality defects. Also, the space is able to not only guide prescription screening and manufacturing process adjustments, but also evaluate the impact of variability in APIs and key excipients on the performance of formulation [12,39,40]. However, current dissolution spaces face a challenge: Can in vitro dissolution methods accurately mirror in vivo release behavior? This is especially problematic for glyburide dissolution. Due to its low solubility, SDS is normally added in in vitro dissolution experiments to create leaky tank conditions, but resulting dissolution curves may not align well with actual in vivo dissolution. Existing studies frequently employ two or three step dissolution set-ups [41,42,43], which adjust the pH of dissolution media and use media with different components. Then, they construct models using mechanistic approaches by deconvoluting key parameters which reflect the release characteristics of formulations. The next study will focus on further refining the model through the integration of more physiological variables affecting in vivo drug dissolution and release to better reflect in vivo release behavior and validate/optimize the dissolution design space.

Currently, BCS-based biowaivers are receiving increasing attention. However, according to regulatory guidelines from various countries and regions, only a subset of BCS Class I and III drugs are eligible for BE waivers, and the criteria are even more stringent for FDC products [44,45]. For drugs that are not eligible for biowaivers, in vivo BE studies remain the gold standard for assessing bioequivalence between test and reference formulations. Nevertheless, the application of VBE and dissolution safe space approaches can provide valuable support in the evaluation of BE for FDCs. In the present study, PBPK model-based sensitivity analysis revealed that glyburide absorption is highly sensitive to gastrointestinal pH, while metformin absorption is more influenced by GI transit time. These findings highlight that intra- and inter-individual variability in GI pH may significantly affect BE outcomes for such combinations. Furthermore, considering the more pronounced fluctuations in GI pH under fed conditions and the clinical recommendation that metformin be administered with food, fed-state BE studies may be more relevant and informative for this FDC. The integration strategy combining the dissolution safe space with a validated PBPK model provides a framework toward establishing robust in vitro–in vivo correlations (IVIVC). In the future, we anticipate that this approach may offer a scientific basis for supporting biowaiver applications in the context of certain minor formulation changes. 

## 5. Conclusions

This study successfully establishes a PBBM for a metformin–glyburide FDC tablet, marking a significant advancement over single-entity modeling approaches. The model uniquely captures the distinct in vivo behaviors of both APIs, accounting for metformin’s solubility-driven and transporter-mediated absorption and glyburide’s pH-dependent dissolution and permeability-limited uptake. By leveraging VBE simulations and the exploration of dual dissolution safe spaces, this work provides a scientifically robust framework to ensure combined BE despite the divergent biopharmaceutical properties of the two drugs.

The significance of this research lies in its ability to broaden the acceptance criteria for traditional in vitro dissolution testing by providing a mechanistic foundation for establishing clinically relevant dissolution specifications. Furthermore, it offers a powerful tool to streamline the development and quality control of metformin–glyburide FDC products, potentially reducing regulatory risk and supporting waiver requests for certain post-approval changes.

Looking forward, future research can focus on prospectively validating the established dissolution safe space with clinical data. Additionally, extending the VBE to specific subpopulations, such as patients with type 2 diabetes who may present altered gastrointestinal physiology, would enhance the model’s clinical relevance and utility. This approach paves the way for more efficient and scientifically grounded development of complex generic and innovative FDC products.

## Figures and Tables

**Table 1:** Physicochemical and biopharmaceutical properties of metformin and glyburide.

Parameters	Unit	Value	Source
Metformin			
Physicochemical properties			
LogP	Log unit	−1.43	[22]
MW	g/mol	129.16	Pubchem
pKa_1_	\	2.8	[23]
pKa_2_	\	11.5	[23]
Solubility (Ref-pH 6.8)	g/L	350.9	[23]
Absorption			
Intestinal permeability (transcellular)	cm/min	5.80 × 10^−8^	optimized
PMAT Hill coefficient	\	2.64	[24]
PMAT Km	μmol/L	1320	optimized
PMAT Vmax	1/min	53.36	optimized
Distribution			
Fraction unbound	%	100	Drugbank
Elimination			
TSmax_spec	μmol/L/min	85.42	optimized
Km(kidney)	μmol/L	65.6	optimized
Formulation			
50% dissolved time	min	9.7	optimized
		10	experiment
Dissolution shape	\	1.37	optimized
		1.22	optimized
Glyburide			
Physicochemical properties			
LogP	Log unit	3.75	Pubchem
MW	g/mol	494	Pubchem
pKa	\	5.3	[25]
Solubility at Reference pH	g/L	2.06 × 10^−3^	Pubchem
Reference pH	\	8.31	optimized
Absorption			
Intestinal permeability (transcellular)	cm/min	6.27 × 10^−5^	optimized
Distribution			
Fraction unbound	%	1	Pubchem
Elimination			
Total hepatic clearance	1/min	4.15	optimized
Formulation			
50% dissolved time	min	77.22	optimized
		82.78	optimized
Dissolution shape	\	1.06	optimized
		1.10	optimized

LogP: Log Partition coefficient, MW: Molecular Weight, pKa_1_: p value of the first acid dissociation constant, pKa_2_: p value of the second acid dissociation constant.

**Table 2:** Predicted and Observed Pharmacokinetic Parameters for Both Drug Components of Reference and Test Formulations, with Fold Errors (FE) and Predict Errors (PE) Estimates.

Compounds	PK Parameters	Formulations	FE	PE
Metformin	AUC_0-t_	T	0.95	0.05
		R	0.97	0.03
	C_max_	T	1.001	0.001
		R	1.07	0.07
Glyburide	AUC_0-t_	T	1.07	0.07
		R	1.13	0.13
	C_max_	T	0.80	0.20
		R	0.80	0.20

**Table 3:** Dissolution Safe Space for Metformin.

Virtual Batch	Dissolution Time (50% Dissolved)	Parameters	T	R	T/R %	90% CI
1	30 min	C_max_ (ng·mL^−1^)	1418.05	1215.67	116.65%	112.75–120.68%
		AUC_0-t_ (ng·h·mL^−1^)	7030.46	5770.53	121.83%	116.62–127.28%
2	25 min	C_max_ (ng·mL^−1^)	1387.88	1215.67	114.17%	110.31–118.15%
		AUC_0-t_ (ng·h·mL^−1^)	6801.63	5770.53	117.87%	112.75–123.21%
3	10 min	C_max_ (ng·mL^−1^)	1263.52	1215.67	103.94%	100.13–107.88%
		AUC_0-t_ (ng·h·mL^−1^)	6099.01	5770.53	105.69%	100.78–110.85%
4	1 min	C_max_ (ng·mL^−1^)	1197.00	1215.67	98.46%	94.64–102.45%
		AUC_0-t_ (ng·h·mL^−1^)	5798.47	5769.15	100.51%	95.63–105.58%

**Table 4:** Dissolution Safe Space for Glyburide.

Virtual Batch	Dissolution Time (50% Dissolved)	Parameters	T	R	T/R %	90% CI
1	175 min	C_max_ (ng·mL^−1^)	55.32	67.44	82.03%	79.16–84.98%
		AUC_0-t_ (ng·h·mL^−1^)	412.71	408.98	100.91%	97.04–104.94%
2	170 min	C_max_ (ng·mL^−1^)	56.01	67.44	83.05%	80.16–86.02%
		AUC_0-t_ (ng·h·mL^−1^)	414.75	408.98	101.41%	97.54–105.43%
3	83 min	C_max_ (ng·mL^−1^)	66.74	67.44	98.98%	95.35–102.75%
		AUC_0-t_ (ng·h·mL^−1^)	413.15	408.98	101.03%	97.34–104.84%
4	35 min	C_max_ (ng·mL^−1^)	65.33	67.44	96.87%	92.75–101.17%
		AUC_0-t_ (ng·h·mL^−1^)	343.75	408.98	84.05%	80.60–87.65%
5	30 min	C_max_ (ng·mL^−1^)	64.19	67.44	95.18%	91.04–99.49%
		AUC_0-t_ (ng·h·mL^−1^)	331.65	408.98	81.09%	77.67–84.66%

## Data Availability

Most data supporting the reported results can be found in the article and Supplementary Materials, but some of the original data are not publicly available due to confidentiality requirements; interested readers may contact the corresponding author for more information.

## Supplementary Materials

The following supporting information can be downloaded at https://www.mdpi.com/article/10.3390/pharmaceutics17101352/s1; Figure S1: In vitro dissolution profiles of reference and test formulations; Table S1: Demographic Characteristics of Subjects in the Human Bioequivalence Study; Table S2: Parameter ranges of gastrointestinal variants in virtual population; Table S3: Virtual Bioequivalence Population Stratification [26,44,46].

## Abbreviations

The following abbreviations are used in this manuscript:

FDC	fixed-dose combination
BE	bioequivalence
API	active pharmaceutical ingredient
PBBM	physiologically based biopharmaceutics modeling
PBPK	physiologically based pharmacokinetic
PK	pharmacokinetics
VBE	virtual bioequivalence
GI	gastrointestinal
MIDD	model-informed drug development
BCS	biopharmaceutics classification system
PMAT	plasma membrane monoamine transporter
FE	fold errors
PE	predict errors
IIV	inter-individual variability
BSV	between-subject variability
WSV	within-subject variability
