# Supplementary material for: Virtual Bioequivalence Assessment and Dissolution Safe Space Exploration for Fixed-Dose Metformin–Glyburide Tablet Using Physiologically Based Biopharmaceutics Modeling
Source: Pharmaceutics. 2025 Oct 20;17(10):1352. doi: 10.3390/pharmaceutics17101352 (PMC12566952; doi:10.3390/pharmaceutics17101352)
Supplement: Supplementary file 1 [file pharmaceutics-17-01352-s001.zip › pharmaceutics-3893212-supplementary.pdf]

pharmaceutics

Supplementary Material

Virtual Bioequivalence Assessment and Dissolution Safe Space Exploration for Fixed-Dose Metformin–Glyburide Tablet using Physiologically Based Biopharmaceutics Modeling

Chenshuang Zhao, Chaozhuang Shen, Yumeng Xiao and Ling Wang*

Department of Clinical Pharmacy and Pharmacy Administration, West China school of Pharmacy, Sichuan University, Chengdu, 610064, China; zhaocs@stu.scu.edu.cn (C.Z.); 2023324050039@stu.scu.edu.cn (C.S.); 2022324050036@stu.scu.edu.cn (Y.X.).

* Correspondence: wlin_scu@scu.edu.cn

S1. Details of In Vitro Dissolution Tests

The selection of these parameters was carefully designed based on the physicochemical properties of the drugs and in accordance with relevant guidelines. The rationale for each condition is provided below:

(1) Volume of Dissolution Medium

The volume of 1000 mL was selected in line with recommendations from the Center for Drug Evaluation (CDE) [44]. This volume adequately fulfills the sink condition for metformin hydrochloride due to its high solubility. For glyburide, which has poor solubility, the sink condition was achieved by adding the surfactant sodium lauryl sulfate (SDS) to the medium.

(2) pH Gradient of the Dissolution Media

The pH gradient was chosen to simulate the physiological environments of the gastrointestinal tract: pH 1.2 represents the fasting stomach; pH 4.5 mimics the gastric environment after emptying or postprandially, as well as the proximal duodenum; and pH 6.8 represents the mildly alkaline environment of the distal small intestine and proximal colon. This range covers the major physiological pH conditions a drug encounters after oral administration and also meets the requirements of CDE guidelines. It is noteworthy that since glyburide is practically insoluble at pH 1.2, its dissolution profiles are provided only for pH 4.5 and 6.8 in the supplementary file.

(3) Sampling Time Points

The sampling time points were chosen to adequately characterize the dissolution profiles of both APIs with differing solubilities and release characteristics. For the highly soluble metformin, time points at 5, 10, 15, 30, 45, and 60 minutes were selected to capture its rapid release. For the poorly soluble glyburide, a longer and more extensive sampling schedule up to 240 minutes (5, 10, 15, 30, 45, 60, 90, 120, 180, and 240 minutes) was implemented to accurately monitor its release from initial onset until a stable plateau exceeding 85% dissolution was reached.

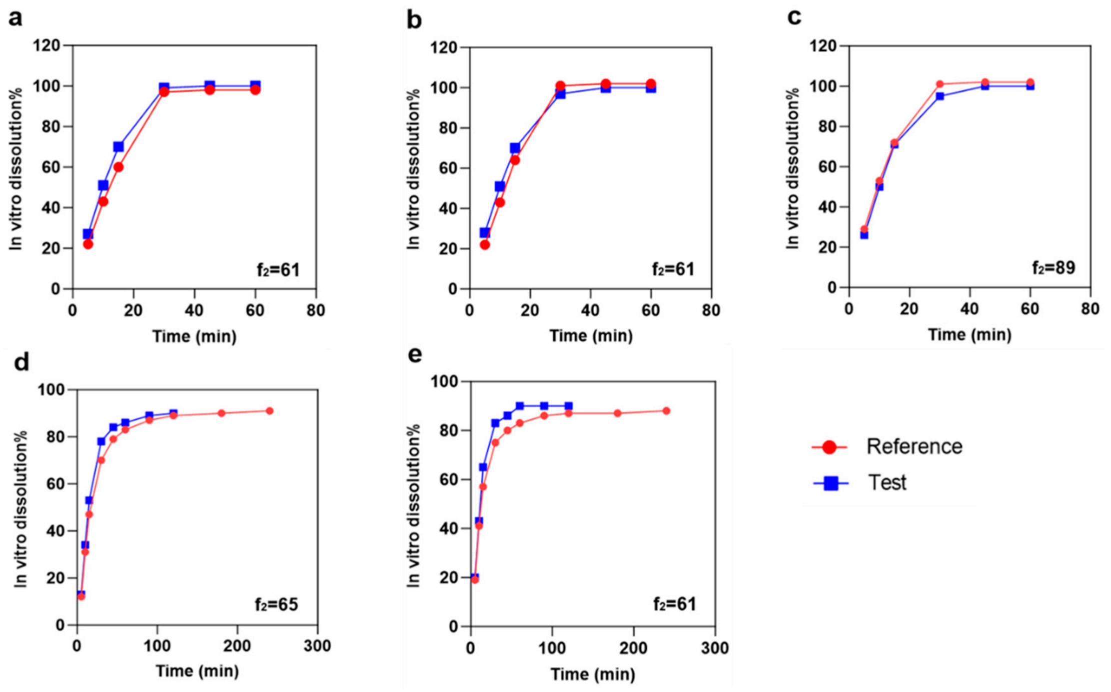

Figure S1. In vitro dissolution profiles of reference (red) and test (blue) formulations: (a-c) represent metformin dissolution under pH 1.2, 4.5, and 6.8 conditions, respectively; (d-e) show glyburide dissolution at pH 4.5 and 6.8 conditions.

S2. 2×2 Cross-Over Study Design

Table S1. Demographic Characteristics of Subjects in the Human Bioequivalence Study.

Population	Chinese
Gender	50% male，50% female
Age	18-60
BMI	19.00-27.00

Table S2. Parameter ranges of gastrointestinal variants in virtual population.

Path	Mean	Deviation	Source
Duodenum pH	5.67	0.61	[46,26]
Lower Jejunum pH	6.51	0.35	[46,26]
Upper Jejunum pH	6.51	0.35	[46,26]
Lower ileum pH	7.42	0.29	[46,26]
Upper ileum pH	7.42	0.29	[46,26]
Colon Sigmoid pH	7.10	0.40	[46,26]
Gastric emptying time (min)	15.00	1.60	mobi
Small intestinal transit time (h)	2.10	1.60	mobi

Table S3. Virtual Bioequivalence Population Stratification.

Allocation	Period Dependent GI Variation	Simulation
Sequence TR	TR-P1 (GI1)	TR-P1-Test
	TR-P2 (GI2)	TR-P2-Reference
Sequence RT	RT-P1 (GI1)	RT-P1-Reference
	RT-P2 (GI2)	RT-P2-Test

T : test；R : reference；P : period；GI : gastrointestinal.
